# Complete Plastid Genome Sequencing of Eight Species from *Hansenia*, *Haplosphaera* and *Sinodielsia* (Apiaceae): Comparative Analyses and Phylogenetic Implications

**DOI:** 10.3390/plants9111523

**Published:** 2020-11-09

**Authors:** Wei Gou, Sheng-Bin Jia, Megan Price, Xian-Lin Guo, Song-Dong Zhou, Xing-Jin He

**Affiliations:** 1 Key Laboratory of Bio-Resources and Eco-Environment of Ministry of Education, College of Life Sciences, Sichuan University, Chengdu 610065, China; gouwei1@stu.scu.edu.cn (W.G.); sdjiashengbin@gmail.com (S.-B.J.); xlguo@stu.scu.edu.cn (X.-L.G.); zsd@scu.edu.cn (S.-D.Z.); 2 Sichuan Key Laboratory of Conservation Biology on Endangered Wildlife, College of Life Sciences, Sichuan University, Chengdu 610065, China; meganprice@scu.edu.cn

**Keywords:** Apiaceae, *Hansenia*, *Haplosphaera*, phylogeny, plastid genome, *Sinodielsia*

## Abstract

*Hansenia* Turcz., *Haplosphaera* Hand.-Mazz. and *Sinodielsia* H.Wolff are three Apiaceae genera endemic to the Hengduan Mountains and the Himalayas, which usually inhabit elevations greater than 2000 m. The phylogenetic relationships between and within the genera were uncertain, especially the placement of *Hap. himalayensis* and *S. microloba*. Therefore, we aimed to conduct comparative (simple sequence repeat (SSR) structure, codon usage bias, nucleotide diversity (Pi) and inverted repeat (IR) boundaries) and phylogenetic analyses of *Hansenia*, *Haplosphaera* and *Sinodielsia* (also compared with *Chamaesium* and *Bupleurum*) to reduce uncertainties in intergeneric and interspecific relationships. We newly assembled eight plastid genomes from *Hansenia*, *Haplosphaera* and *Sinodielsia* species, and analyzed them with two plastid genomes from GenBank of *Hap. phaea,* *S. yunnanensis*. Phylogenetic analyses used these ten genomes and another 22 plastid genome sequences of Apiaceae. We found that the newly assembled eight genomes ranged from 155,435 bp to 157,797 bp in length and all had a typical quadripartite structure. Fifty-five to 75 SSRs were found in *Hansenia*, *Haplosphaera* and *Sinodielsia* species, and the most abundant SSR was mononucleotide, which accounted for 58.47% of *Hansenia*, 60.21% of *Haplosphaera* and 48.01% of *Sinodielsia.* There was no evident divergence of codon usage frequency between the three genera, where codons ranged from 21,134 to 21,254. The Pi analysis showed that *trnE(UUC)-trnT(GGU)*, *trnH(GUG)-psbA* and *trnE(UUC)-trnT(GGU)* spacer regions had the highest Pi values in the plastid genomes of *Hansenia* (0.01889), *Haplosphaera* (0.04333) and *Sinodielsia* (0.01222), respectively. The *ndhG-ndhI* spacer regions were found in all three genera to have higher diversity values (Pi values: 0.01028–0.2), and thus may provide potential DNA barcodes in phylogenetic analysis. IR boundary analysis showed that the length of *rps19* and *ycf1* genes entering IRs were usually stable in the same genus. Our phylogenetic tree demonstrated that *Hap. himalayensis* is sister to *Han. weberbaueriana*; meanwhile, *Haplosphaera* and *Hansenia* are nested together in the East Asia clade, and *S. microloba* is nested within individuals of *S. yunnanensis* in the *Acronema* clade. This study will enrich the complete plastid genome dataset of the Apiaceae genera and has provided a new insight into phylogeny reconstruction using complete plastid genomes of *Hansenia*, *Haplosphaera* and *Sinodielsia*.

## 1. Introduction

**Figure 1:**
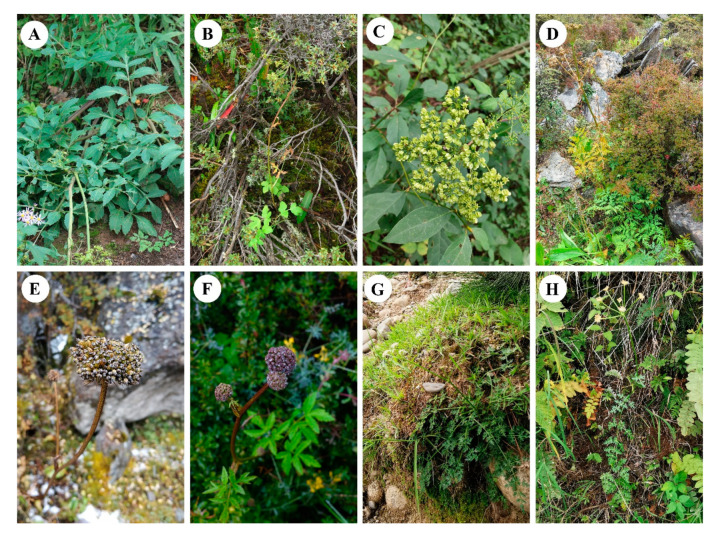
Plants of *Hansenia*, *Haplosphaera* and *Sinodielsia*. (**A**) *Han. forbesii*, (**B**) *Han. forrestii*, (**C**) *Han. oviformis*, (**D**) *Han. weberbaueriana*, (**E**) *Hap. himalayensis*, (**F**) *Hap. phaea*, (**G**) *S. microloba* and (**H**) *S. yunnanensis*.

*Hansenia* Turcz., *Haplosphaera* Hand.-Mazz. and *Sinodielsia* H.Wolff are three endemic high-elevation (typically >2000 m) genera of Apiaceae, mainly distributed in the Hengduan Mountains and the Himalayas (Figure 1) [1]. According to the latest Apiaceae taxonomy, *Hansenia*, *Haplosphaera* and *Sinodielsia* comprise five, two and four species, respectively [2,3,4,5]. Previous phylogenetic studies of *Hansenia*, *Haplosphaera* and *Sinodielsia* based on two plastid genome regions (*rpl16* and *rps16* introns) and nuclear internal transcribed spacers (nrITSs) found that *Sinodielsia* is within the *Acronema* clade, while the closely related *Hansenia* and *Haplosphaera* are located in the East Asia clade, albeit from limited sampling [6,7,8,9,10,11]. One study of 106 nrITS sequences representing 100 species from 52 genera of Chinese Apiaceae found that the *Acronema* clade and the East Asia clade were well-supported (posterior probability both valued 100% by Bayesian inference) [8]. Additionally, *Hap. himalayensis* and *S. microloba* were unknown at the time of these previous studies and remain as two little-known species.

With the development of second-generation sequencing technology, more plastid genomes have been used in phylogeny and comparative studies, and fairly good results have been obtained [12]. Generally the circular genome consists of two inverted repeats (IRs) divided by two regions, the large (LSC) and small single-copy (SSC) regions [13,14], and most angiosperm complete plastid genomes are between 115 and 165 kb in length [15]. The gene content and order of plastid genomes are usually highly conserved, and the substitution rate in plastid DNA is much lower than in plant nuclear DNA [16]. The similarity of gene length and the low substitution rate of plant plastids make them valuable sources of genetic markers for phylogenetic studies [17].

In our previous studies, we sequenced two plastid genomes of *Hap. phaea* [18] and *S. yunnanensis* (HB: Zhongdian population) [19] and provided preliminary phylogenetic positions for the two species. Following on from this, we aimed to conduct comparative (simple sequence repeat (SSR), codon usage bias, nucleotide diversity (Pi) and IR) and phylogenetic analyses of *Hansenia*, *Haplosphaera* and *Sinodielsia* to reduce uncertainties in intergeneric and interspecific relationships. Analyses were conducted using a 32 complete plastid genome dataset, which was compiled from eight newly assembled plastid genomes from *Han. forbesii*, *Han. forrestii*, *Han. oviformis*, *Han. weberbaueriana*, *Hap. himalayensis*, *S. microloba*, *S. yunnanensis* (EY: Eryuan County pop.) and *S. yunnanensis* (KM: Kunming pop.), as well as previously sequenced genomes. Our study provides newly complete plastid genomes of *Hansenia*, *Haplosphaera* and *Sinodielsia* species, and since these three genera are endemic to Pan-Himalayan regions, the information provided herein is indispensable for Apiaceae plastid evolutionary and phylogenetic studies.

## 2. Results and Discussion

### 2.1. Phylogenetic Analysis

**Figure 2:**
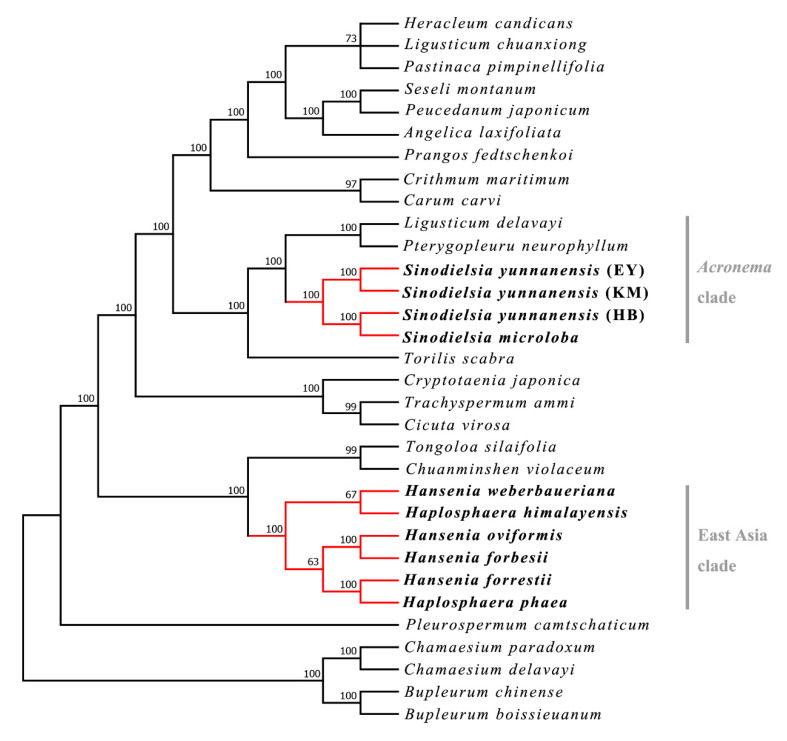
Maximum Likelihood (ML) phylogenetic tree of Apiaceae using the 32 plastid genomes dataset. The studied taxa are bold. The numbers above the nodes are Maximum Likelihood-Bootstrap Support (ML-BS) presented as percentages (>50%). The names of the clades follow the study of Zhou et al. [8,9].

The phylogenetic tree (Figure 2) showed that *Hansenia* and *Haplosphaera* form a strongly supported monophyly (Maximum Likelihood-Bootstrap Support (ML-BS) = 100%) in the East Asia clade. *Hap. himalayensis* is sister to *Han. weberbaueriana* (ML-BS = 67%), and *Hap. phaea,* the type species of *Haplosphaera*, is sister to *Han. forrestii* (ML-BS = 100%), showing that *Haplosphaera* is nested within *Hansenia*. This suggests that *Hansenia* and *Haplosphaera* should be combined to a single genus, as already proposed in previous phylogenetic studies. [8,9,20].

The tree also showed that *S. microloba* is sister to *S. yunnanensis* (HB) (ML-BS = 100%) and allied with other *S. yunnanensis* (EY and KM) (ML-BS = 100%) populations instead of other species in the *Acronema* clade. The extremely close distance between *S. microloba* and *S. yunnanensis* is unusual compared to other Apiaceae species, even though they are congeneric [21,22,23]. However, their morphological characters are distinctive (Figure 1). We speculate there may have been a hybridization phenomenon (i.e., recent or ongoing) between the two species or other intrageneric species, which have to also use nuclear markers to disentangle the true phylogeny of the species. Otherwise, the observed closeness may be caused by incomplete lineage sorting. More populations of *S. microloba* and other *Sinodielsia* species are needed to explore their relationship and evolution.

### 2.2. The Plastid Genomes of Hansenia, Haplosphaera and Sinodielsia Species

**Figure 3:**
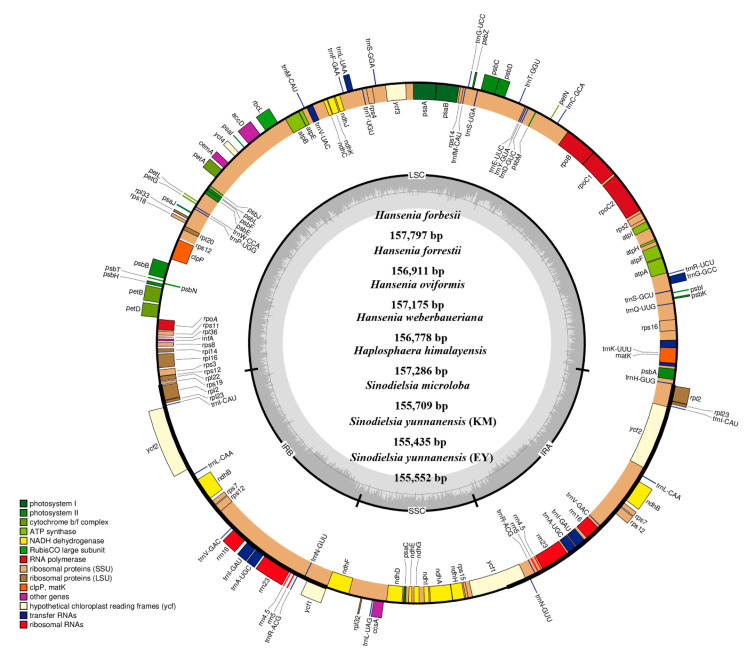
Plastid genome map of eight *Hansenia*, *Haplosphaera* and *Sinodielsia* species (for a better view, these eight maps were combined into one derived from the map of *H. forbesii* because they have the same order and composition of genes). The genes shown inside and outside of the circle indicate those transcribed in the clockwise and counterclockwise direction, respectively. Genes of different functional groups are colored differently. The GC contents are shown in the inner circle with darker grey.

The complete plastid genomes of *Hansenia, Haplosphaera* and *Sinodielsia* species exhibited a typical quadripartite organization of a single circular DNA molecule (Figure 3). The lengths of the ten genomes (four *Hansenia,* two *Haplosphaera* and four *Sinodielsia* species, including the additional two populations of *S. yunnanensis*) ranged from 154,670 bp (*S. yunnanensis*: HB) to 157,797 bp (*Han. forbesii*) (Table 1). Two identical IRs (including IRa and IRb, with lengths 26,404–26,542 bp) were found in the plastid genomes, which were separated by LSC (85,233–86,968 bp) and SSC (17,370–17,891 bp) regions. The quadripartite organization was found in most plastid genomes of higher plants [13,14], while IRs were absent in *Taxus chinensis* var. *mairei*, *Erodium* species, *Pisum sativum* and *Vicia faba* [21,24,25], which may lead to a reduction in the number of duplicated genes and the length of whole plastid genomes. In Apiaceae, the IRa and IRb were both present in the studied plastid genomes from GenBank, *Bupleurum* species [26] and *Chamaesium* species [27] ranging from 26,280–26,303 bp and 25,727–26,147 bp, respectively, and the difference of length is mainly caused by the loss or insertion of the spacer regions. The *Hansenia*, *Haplosphaera* and *Sinodielsia* plastid genomes had almost identical GC content (37.5–37.7%) to whole plastid genomes. Higher GC content (42.7–42.8%) was detected in the IR regions compared to the average of a whole genome, which was possibly due to the presence of rRNA sequences with high GC content (55.2–55.3%) in IR regions. Similarly, the high GC content of rRNA sequences also occurs in other Apiaceae species [26,27]. The ten new plastid genomes contained 133 genes, including eight rRNA, 37 tRNA and 85 protein-coding genes (PCGs) (Table 2). Among these 133 genes, 95 genes only had one copy, while 19 genes were duplicated in the IRa and IRb regions, including four rRNA genes (*rrn4.5*, *rrn5*, *rrn16* and *rrn23*), seven tRNA genes (*trnA-UGC*, *trnI-CAU*, *trnI-GAU*, *trnL-CAA*, *trnN-GUU*, *trnR-ACG* and *trnV-GAC*) and eight PCGs (*ndhB*, *rpl2*, *rpl23*, *rps7*, *rps12*, *rps19*, *ycf1* and *ycf2*). Four pseudogenes—*ψrps19*, *ψycf1* and two *ψycf15*—were found in all the ten genomes. In comparison, *Hansenia, Haplosphaera, Sinodielsia*, *Bupleurum* [26] and *Chamaesium* [27] plastid genes are consistent in their total number of predicted coding regions.

The PCGs in the *Hansenia*, *Haplosphaera* and *Sinodielsia* plastid genomes included five genes (*psaA*, *psaB*, *psaC*, *psaI* and *psaJ*) encoding photosystem I subunits, while 15 genes (*psbA*, *psbB*, *psbC*, *psbD*, *psbE*, *psbF*, *psbH*, *psbI*, *psbJ*, *psbK*, *psbL*, *psbM*, *psbN*, *psbT* and *psbZ*) were related to photosystem II subunits. Nine (*rpl2*, *rpl14*, *rpl16*, *rpl20*, *rpl22*, *rpl23*, *rpl32*, *rpl33* and *rpl36*) encoding large ribosomal protein genes and 12 (*rps2*, *rps3*, *rps4*, *rps7*, *rps8*, *rps11*, *rps12*, *rps14*, *rps15*, *rps16*, *rps18* and *rps19*) encoding small ribosomal protein genes were detected. Additionally, six genes (*atpA*, *atpB*, *atpE*, *atpF*, *atpH* and *atpI*) of ATP synthase subunits were detected. These PCGs were also detected in *Bupleurum* [26] and *Chamaesium* species [27] and are generally involved in the important process of plant growth.

### 2.3. IR Boundaries and Simple Sequence Repeats (SSRs) Structure Analysis

**Figure 4:**
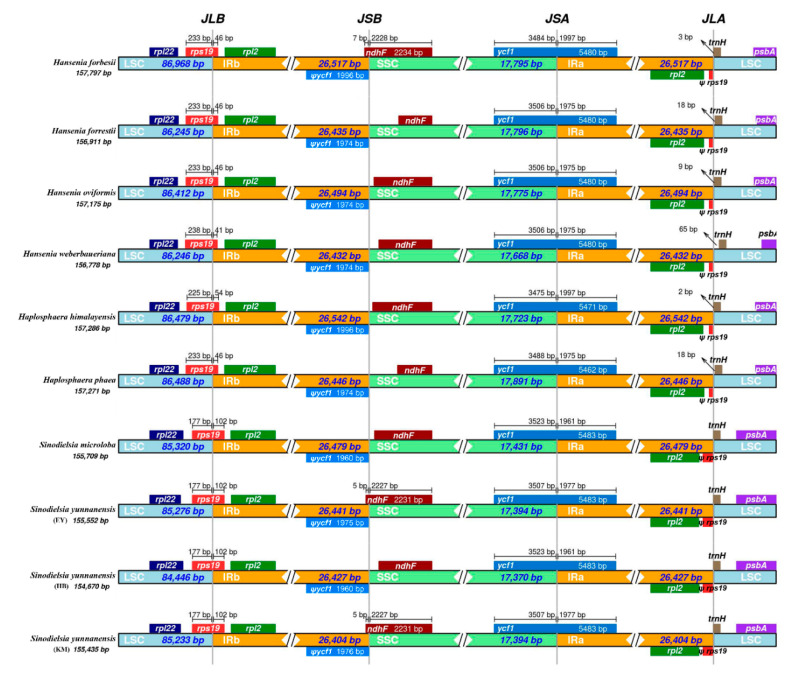
Comparisons of inverted repeat (IR) boundaries between ten *Hansenia*, *Haplosphaera* and *Sinodielsia* plastid genomes.

The IR boundaries of the ten *Hansenia*, *Haplosphaera* and *Sinodielsia* plastid genomes were compared to analyze the fluctuations (expansion or contraction) in these regions (Figure 4). Although the ten plastid genomes showed a similar structure and content, some variations were still identified. The *rps19* gene entered the IRb region with 46 bp, 46 bp, 46 bp, 41 bp, 54 bp and 46 bp in the plastid genomes of *Han. forbesii*, *Han. forrestii*, *Han. oviformis*, *Han. weberbaueriana*, *Hap. himalayensis* and *Hap. phaea* (respectively), while 102 bp, 102 bp, 102 bp and 102 bp were entered in the plastid genomes of *S. microloba*, *S. yunnanensis* (EY), *S. yunnanensis* (HB) and *S. yunnanensis* (KM), respectively. The *ndhF* genes of *Han. forrestii*, *Han. oviformis*, *Han. weberbaueriana*, *Hap. himalayensis*, *Hap. phaea*, *S. microloba* and *S. yunnanensis* (HB) are entirely within the SSC region, and a 6–48 bp intergenic region exists between the *ndhF* gene and the JSB line (the border between IRb and SSC), while the *ndhF* genes of *Han. forbesii*, *S. yunnanensis* (EY) and *S. yunnanensis* (KM) are partly included in IRb regions with 7 bp, 5 bp and 5 bp, respectively. The *ycf1* gene of the ten plastid genomes occupies the JSA line (the border between SSC and IRa), with a length ranging from 1961 to 1997 bp in IRa regions, and ranging from 3475 to 3523 bp in close SSC regions. This also created a pseudogene *ycf1* in the IRb regions. The *trnH* genes of *Han. forbesii*, *Han. forrestii*, *Han. oviformis*, *Han. weberbaueriana*, *Hap. himalayensis* and *Hap. phaea* are entirely within the LSC region. A 2–65 bp length of the intergenic region exists between the *trnH* genes and JLA line (the border between IRa and SSC), while the *trnH* gene of *S. microloba*, *S. yunnanensis* (EY), *S. yunnanensis* (HB) and *S. yunnanensis* (KM) is included in IRa regions with 1 bp.

We found that the lengths of *rps19* and *trnH* genes entering IRs are similar between *Hansenia* (36–41 bp) and *Haplosphaera* (41–49 bp), which is different from these genes in *Sinodielsia* (102 bp). Not surprisingly, *Hansenia* is more closely related to *Haplosphaera* but distantly related to *Sinodielsia*, which has also been confirmed by nrITS sequences and transcriptome data [8,20]. A collective analysis of our results and previous studies [26,27,28,29] found that the lengths of *rps19* and *trnH* genes entering IRs are usually stable in the same genus or phylogenetically related groups. For example, the length of *rps19* gene entering IRs is 57–96 bp in *Chamaesium* species [27], and 49–84 bp in *Bupleurum* species [26], which are the phylogenetically basal groups in the *Chamaesium* clade and Bupleureae. Whereas in Apieae members, such as *Anethum*, *Apium* and *Petroselium*, the *rps19* genes are not in the IRs. Instead, the *rpl2* genes are partly in the IRs [26,30]. Although the *rpl2* genes are partly duplicated in the IRs of some Apieae members, they are duplicated completely in the *Hansenia*, *Haplosphaera* and *Sinodielsia* species. As for the two pseudogenes *ψrps19* and *ψycf1* detected in the plastid genomes of *Hansenia*, *Haplosphaera* and *Sinodielsia*, their incomplete duplication may be caused by the fluctuations in the IR boundary. The fluctuations may lead to gene loss (or pseudogene loss), which is a common phenomenon in Apiaceae species [26,27]. Nevertheless, the length of land plants IRs can vary from 10 to 76 kbp, with most species having an IR of about 25 kbp, and often less than 15 kbp in lower land plants and fern species [31]. Fluctuations in IR regions are the main reason for the differences in plastid genome lengths in most species, which also leads to several genes entering the IR regions or the single-copy sequences [32].

**Figure 5:**
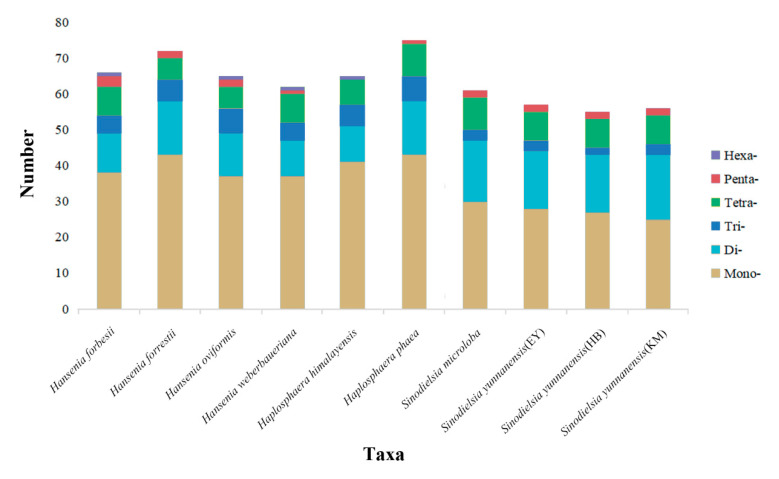
Frequency of detected simple sequence repeat (SSR) motifs in different repeat types in ten *Hansenia*, *Haplosphaera* and *Sinodielsia* plastid genomes.

**Figure 6:**
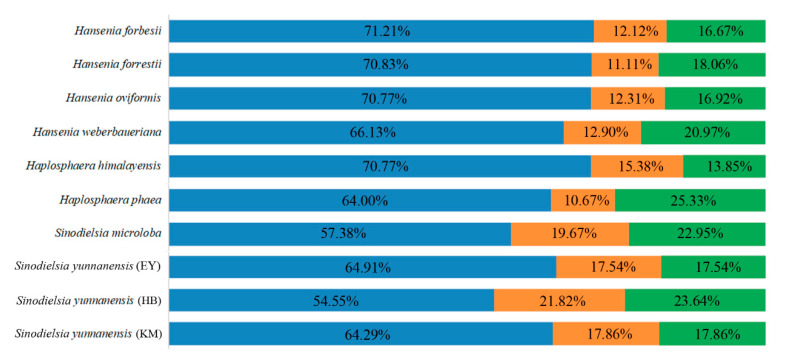
Frequency of detected SSR motifs in LSC (blue), IR (orange) and SSC (green) regions of the ten *Hansenia*, *Haplosphaera* and *Sinodielsia* plastid genomes.

Sixty-six, 72, 65, 62, 65, 75, 61, 57, 55 and 56 SSRs were identified in the plastid genomes of *Han. forbesii*, *Han. forrestii*, *Han. oviformis*, *Han. weberbaueriana*, *Hap. himalayensis*, *Hap. phaea*, *S. microloba*, *S. yunnanensis* (EY), *S. yunnanensis* (HB) and *S. yunnanensis* (KM), respectively. The results hint that plastids of related groups share similar numbers of SSRs, while that is not always the result [26]. Six repeat motifs (mono-, di-, tri-, tetra-, penta- and hexanucleotide repeats) of microsatellites were detected in the plastid genome of the *Hansenia*, *Haplosphaera* and *Sinodielsia* species. Mononucleotide repeats were the most abundant SSR, which accounted for 58.47% of *Hansenia*, 60.21% of *Haplosphaera* and 48.01% of *Sinodielsia*. Dinucleotide SSRs were the second most abundant SSR, which accounted for 18.02% of *Hansenia*, 17.69% of *Haplosphaera* and 29.29% of *Sinodielsia*. This was followed by tetranucleotide repeats of 8.69%, 9.28% and 4.79%, and trinucleotide repeats of 10.65%, 11.38% and 14.41% in *Hansenia, Haplosphaera* and *Sinodielsia*, respectively. Pentanucleotide and hexanucleotide were the least abundant SSR (average of the three genera: 2.73% and 0.62%, respectively) (Figure 5). The repeat motifs with the highest content in SSRs were all mononucleotide, which is similar to most species including Apiaceae plants [26,27], but different from *Forthysia* (dinucleotide) [33] and *Nitotiana* (trinucleotide) species [34]. The similar contents of mononucleotide and dinucleotide repeats of *Hansenia* and *Haplosphaera* indicates that both genera seem to belong together. In all *Hansenia*, *Haplosphaera* and *Sinodielsia* species the repeats were composed almost entirely of A/T, except for mononucleotide repeat motifs that also had G/C contents of 10.53%, 2.33%, 10.81%, 7.32% and 4.65 in *Han. forbesii*, *Han. forrestii*, *Han. weberbaueriana*, *Hap. himalayensis* and *Hap. phaea*. The phylogenetic closeness of these species suggests that the relatives may share similar mononucleotide repeated contents. Most dinucleotide repeats were AT/TA, except for one TC repeat that was in the plastid genome of *S. yunnanensis* (HB). Across all SSR loci, 94 SSRs (14.83%) were found in the IRs, 417 SSRs (65.77%) in LSC regions and 123 SSRs (19.40%) in SSC regions of the plastid genomes (Figure 6).

### 2.4. Codon Usage Analysis

**Figure 7:**
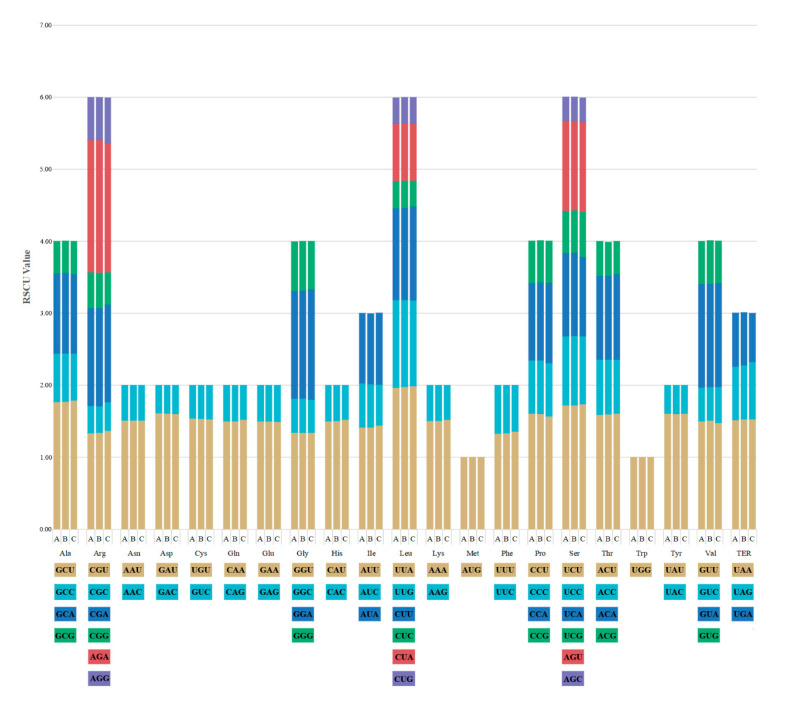
Codon content of 20 amino acids and the stop codon present in all 53 studied protein-coding genes (PCGs) of the (**A**) *Hansenia*, (**B**) *Haplosphaera* and (**C**) *Sinodielsia* plastid genome. Different colors of the histogram correspond to the different codons below.

The codon usage bias and relative synonymous codon usage (RSCU) values were calculated using 53 PCGs in the ten *Hansenia*, *Haplosphaera* and *Sinodielsia* plastid genomes. There was no evident divergence of the codon usage frequency when we compared the three genera (Figure 7). The number of codons of PCGs ranged from 21,134 (*S. yunnanensis*: KM) to 21,254 (*Han. weberbaueriana*) (Supplementary Table S2). Among these codons, leucine was encoded by 2227–2236 and cysteine was encoded by 214–224 codons, which presented the maximum and the minimum number of codons per amino acids in our study species, respectively. AUU (850–872) involved in encoding isoleucine and UAG (13–14) involved in encoding a terminator were the most and least used codons. Furthermore, 30 codons of *Hansenia* and *Haplosphaera*, and 31 codons of *Sinodielsia* plastid genomes had RSCU values larger than 1, indicating that they were the preferred codons in those ten plastid genomes. Among these 30/31 preferred codons, most codons terminated in A/T, except that UUG ended with G, and C was not found at the third position. This demonstrated that the codon usages of the ten plastid genomes were biased towards A/T at the third position of codons, which is generally consistent with other reported genomes of angiosperm [35,36], including the *Bupleurum* [26] and *Chamaesium* [27] species.

Codon usage bias is an important indicator for studying the evolutionary relationship of genomes [37]. Studies have shown that many biological factors affect the preference of synonymous codon usage, such as gene expression level [38], gene sequence length [39], tRNA abundance [40] and GC distribution position [41]. Codon bias seems to be maintained by selection–mutation–drift balance [42]. There is a general agreement that the strong bias towards highly expressed genes is due to selection for speed or translational efficiency [43]. Our studied species of *Hansenia*, *Haplosphaera* and *Sinodielsia* are from the Himalayan and Hengduan regions, usually inhabiting alpine areas over 3000 m, and one of the reasons for their similar RSCU values may be the shared natural selection pressures, even though they are not all closely related. This may provide another analytical technique to use when studying nuclear genomes and can assist in understanding how species of separate lineages, yet similar environments, have undergone parallel evolution. Increased numbers and improved methods of analytical techniques are especially useful when studying many Pan-Himalayan region Apiaceae species that are morphologically similar.

### 2.5. Nucleotide Diversity Analysis

**Figure 8:**
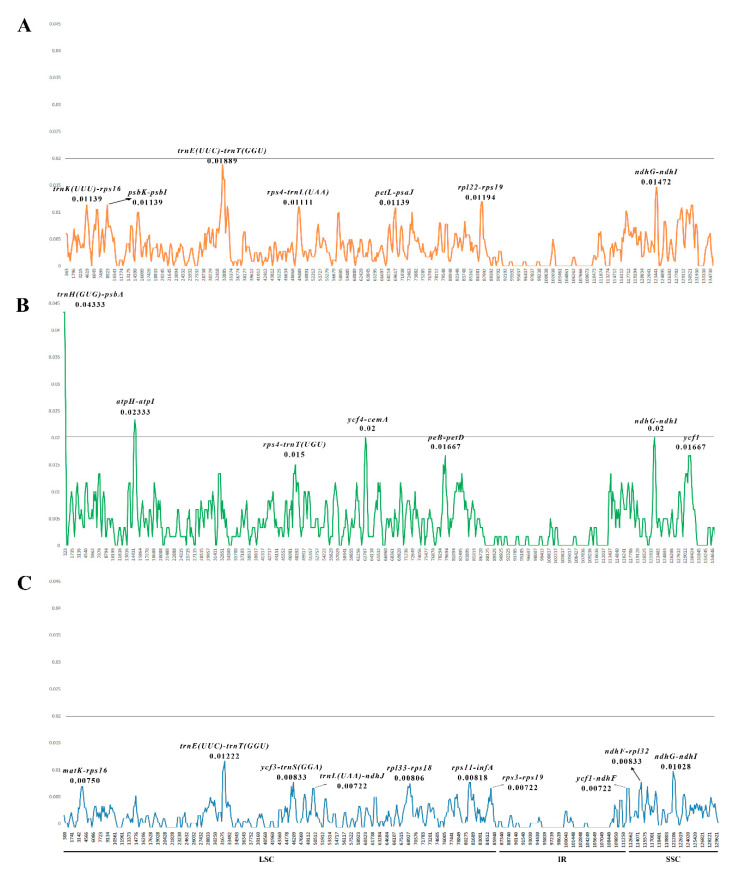
The nucleotide diversity of (**A**) the four *Hansenia*, (**B**) the two *Haplosphaera* and (**C**) the four *Sinodielsia* plastid genomes. The positions of 0.02 in all three graphs were marked by a line. Ten or seven regions with the highest Pi values were named out.

Nucleotide diversity (Pi) values of the plastid genomes from the *Hansenia*, *Haplosphaera* and *Sinodielsia* species were calculated to evaluate their sequence divergence level (Figure 8). In the four *Hansenia* genomes, Pi values in the LSC regions ranged from 0 to 0.01889, with an average of 0.00371, and ranged from 0 to 0.01472 in the SSC regions, with an average of 0.00263. The Pi values of IR regions ranged from 0 to 0.00639 and had an average of 0.00089, the lowest Pi values of the three regions for these four genomes. In the two *Haplosphaera* genomes, Pi values ranged from 0 to 0.04333 and averaged 0.00531 in the LSC regions, and from 0 to 0.02 in the SSC regions, with an average value of 0.0028. The IR region values in the two *Haplosphaera* genomes were similarly low, with an average of 0.0015 and ranging from 0 to 0.01333. The two *Haplosphaera* species with high Pi values seem to be distant species within the genus *Hansenia* (see further under phylogenetic studies). In the two *Sinodielsia* genomes, Pi values ranged from 0 to 0.01222 with an average of 0.0021 in the LSC regions, and from 0 to 0.01028, averaging 0.00149 in the SSC regions. The Pi values of IR regions were again low for the two *Sinodielsia* genomes and ranged from 0 to only 0.00833, with a value of 0.00112. Pi values indicated mutations in the respective regions [26]. The Pi values of *Sinodielsia* were the lowest of the three genera, indicating that *S. microloba* is more closely related to *S. yunnanensis* than the other species within each genus.

We found that high Pi values of sequences were usually detected in spacer regions between genes. Among these genomic spacer regions of the *Hansenia* species, *trnK (UUU)-rps16*, *psbK-psbI*, *trnE (UUC)-trnT (GGU)*, *rps4-trnL (UAA)*, *petL-psaJ*, *rpl22-rps19* and *ndhG-ndhI* had the highest Pi values, ranging from 0.01083 to 0.01889. The highest Pi values of spacer regions of the *Haplosphaera* species ranged between 0.015 and 0.04333 for *trnH (GUG)-psbA*, *atpH-atpI*, *rps4-trnT (UGU)*, *ycf4-cemA*, *petB-petD* and *ndhG-ndhI*. Within the spacer regions of *Sinodielsia* species, *matK-rps16*, *trnE (UUC)-trnT (GGU)*, *ycf3-trnS (GGA)*, *trnL (UAA)-ndhJ*, *rpl33-rps18*, *rps11-infA*, *rps3-rps19*, *ycf1-ndhF*, *ndhF-rpl32* and *ndhG-ndhI* had the highest Pi values, ranging from 0.00722 to 0.01222. Although the spacer regions were diverse among the three genera, we found the spacer regions *ndhG-ndhI* occurred in all three genera with relatively high values. Pending more specimen samples and plastid genome studies of Chinese Apiaceae, the spacer regions (e.g., high Pi value *ndhG-ndhI*) may provide DNA barcodes for molecular identification and phylogenetic studies in the large-scale clades such as East Asia clade and *Acronema* clade, where there are many parallel branches whose support rate is less than 50% using ITS or plastid DNA introns (*rpl16* and *rps16* genes) [8,9].

## 3. Materials and Methods

### 3.1. Material, DNA Extraction and Complete Genome Sequencing

The materials of *Han. forbesii*, *Han. forrestii*, *Han. oviformis*, *Han. weberbaueriana*, *Hap. himalayensis*, *S. microloba, S. yunnanensis* (EY) and *S. yunnanensis* (KM) were newly obtained for this study, which were collected from the type localities or their adjacent areas during 2018–2019 (Supplementary Table S1). The different populations of *S. yunnanensis* are represented by EY: from Eryuan County, Yunnan; KM: from Kunming, and Yunnan; HB: from Zhongdian, Yunnan (HB previously sequenced [19]). The total genomic DNA was extracted from silica gel-dried leaves according to the protocols of the plant genomic DNA kit (cwbio, Beijing, China), then sequenced using the Illumina Novaseq 6000 platform (Illumina, San Diego, CA, USA) with Novaseq 150 sequencing strategy by Novogene (Beijing, China).

### 3.2. Genome Construction and Annotation

The clean data (removed connectors and low-quality reads) were assembled using NOVOPlasty 2.7.1 [44] with K-mer 39, where the *rbcL* gene of *Han. oviformis* and *S. yunnanensis* (amplificated and sequenced beforehand) was used as a seed input and the reference sequence. The assembled complete plastid genomes were checked then aligned with the reference plastid genome of *Chuanminshen violaceum* (KU921430) using GENEIOUS R11 [45] to select the best option, which was then annotated using PGA [46]. GENEIOUS R11 was then used to manually adjust the annotation for uncertain start and stop codons based on the comparison with homologous genes from other annotated plastid genomes. The eight annotated plastid genomes were submitted to GenBank, and their accession numbers are in Supplementary Table S1. Their genome maps were drawn using OGDRAW version 1.3.1 [47].

### 3.3. Phylogenetic Analysis

To better infer phylogenetic relationships between *Hansenia*, *Haplosphaera* and *Sinodielsia*, the 32 plastid genomes were applied for reconstructing the phylogenetic tree, of which 24 plastid genomes were obtained from GenBank (Supplementary Table S1). All the plastid genomes were aligned using MAFFT v7.308 [48,49]. Maximum Likelihood was then conducted for phylogenetic analyses using RAxML version 8.2.4 [50] under the model GTR+G with 1000 rapid bootstraps. The scientific names of plants followed the International Plant Names Index (https://www.ipni.org). *Chamaesium* and *Bupleurum* species were selected as outgroups [8,9].

### 3.4. IRs Boundaries and SSR Analysis

The boundaries between the LSC, SSC and IR regions of the ten plastid genomes were compared and drawn using the online program: IRscope (https://irscope.shinyapps.io/irapp/) [51]. The SSRs were identified using MISA [52] with the repeat threshold settings: 10 repeats for mono-nucleotide, 5 for di-nucleotide, 4 for tri-nucleotide and 3 repeats for tetra-, penta-and hexanucleotide SSRs.

### 3.5. Codon Usage Bias Analysis

Codon usage bias analysis and calculation of RSCU values were conducted using the program CodonW [53]. Fifty-three PCGs (more than 300 bp in length) of each *Hansenia*, *Haplosphaera* and *Sinodielsia* plastid genomes were filtered. The codon adaptation index (CAI), the codon bias index (CBI), the effective number of codons (ENC), the frequency of optimal codons (Fop) and the GC content of the synonymous third codons positions (GC3s) were calculated to assess the extent of the codon usage bias. The RSCU values of the four *Hansenia*, two *Haplosphaera* and four *Sinodielsia* plastid genomes were also calculated to assess their codon usages, including *Hap. phaea* and *S. yunnanensis* (HB) downloaded from GenBank (Supplementary Table S1).

### 3.6. Nucleotide Diversity Analysis

The plastid genomes of *Hansenia*, *Haplosphaera* and *Sinodielsia* species were aligned using MAFFT v7.308 [48,49]. DNA polymorphism analysis was then conducted to calculate the Pi values in DnaSP v5 [54] in the sliding window. The setting parameters were as follows: (1) windows length: 600 sites; (2) step size: 200 sites.

## 4. Conclusions

In this study, the plastid genomes of *Han. forbesii*, *Han. forrestii*, *Han. oviformis*, *Han. weberbaueriana*, *Hap. himalayensis*, *S. microloba*, *S. yunnanensis* (KM) and *S. yunnanensis* (EY) were newly assembled and annotated. All the eight plastid genomes exhibited a typical circular quadripartite organization with similar whole length (155,435 bp to 157,797 bp) and gene contents. The IR boundary analysis showed that length of *rps19* and *ycf1* genes entering IRs are usually stable in the same genus. *Hansenia* shared relatively similar mononucleotide SSRs to *Haplosphaera*. Additionally, *Hansenia*, *Haplosphaera* and *Sinodielsia* species had similar codon usage. Although *Hansenia* and *Haplosphaera* are not phylogenetically close to *Sinodielsia*, there was no significant difference in their plastid genomes. Furthermore, we found that the *ndhG-ndhI* spacer regions possessed higher nucleotide diversity in the three genera and, therefore, may provide DNA barcodes for intra- and inter-genus identification in Apiaceae. The phylogeny of the 32 plastid genomes, including the eight taxa mentioned above, showed a close relationship between *Hansenia* and *Haplosphaera*, and *S. microloba* may be a species of hybrid origin. This study will enrich the complete plastid genome dataset of the genera *Hansenia*, *Haplosphaera* and *Sinodielsia*, and has provided a new insight into comparisons of distant taxa and phylogeny reconstruction using complete plastid genomes of Apiaceae.

## Figures and Tables

**Table 1:** The features of plastid genomes of eight *Hansenia*, *Haplosphaera* and *Sinodielsia* species (IRs: inverted repeats; LSC: large single-copy region; SSC: small single-copy region).

Taxa	Size (bp)	LSC Length (bp)	IR Length (bp)	SSC Length (bp)	Total Genes	Protein Coding Genes	tRNA Genes	rRNA Genes	Overall GC Content (%)
*Hansenia forbesii*	157,797	86,968	26,517	17,795	131	85	37	8	37.6
*Hansenia forrestii*	156,911	86,245	26,435	17,796	131	85	37	8	37.6
*Hansenia oviformis*	157,175	86,412	26,494	17,775	131	85	37	8	37.6
*Hansenia weberbaueriana*	156,778	86,246	26,432	17,668	131	85	37	8	37.7
*Haplosphaera himalayensis*	157,286	86,479	26,542	17,723	131	85	37	8	37.6
*Haplosphaera phaea*	157,271	86,488	26,446	17,891	131	85	37	8	37.6
*Sinodielsia microloba*	155,709	85,320	26,479	17,431	131	85	37	8	37.5
*Sinodielsia yunnanensis* (EY)	155,552	85,276	26,441	17,394	131	85	37	8	37.6
*Sinodielsia yunnanensis* (HB)	154,670	84,446	26,427	17,370	131	85	37	8	37.6
*Sinodielsia yunnanensis* (KM)	155,435	85,233	26,404	17,394	131	85	37	8	37.5

**Table 2:** List of genes encoded in the ten *Hansenia*, *Haplosphaera* and *Sinodielsia* plastid genomes.

Category	Group of Genes	Name of Genes
Self-replication	transfer RNAs (tRNAs)	trnA-UGC *, trnC-GCA, trnD-GUC, trnE-UUC, trnF-GAA, trnfM-CAU, trnG-GCC, trnG-UCC, trnH-GUG, trnI-CAU *, trnI-GAU *, trnK-UUU, trnL-CAA *, trnL-UAA, trnL-UAG, trnM-CAU, trnN-GUU *, trnP-UGG, trnQ-UUG, trnR-ACG *, trnR-UCU, trnS-GCU, trnS-GGA, trnS-UGA, trnT-GGU, trnT-UGU, trnV-GAC *, trnV-UAC, trnW-CCA, trnY-GUA
	ribosomal RNAs (rRNAs)	rrn4.5 *, rrna5 *, rrn16 *, rrn23 *
	RNA polymerase	*rpoA, rpoB, rpoC1, rpoC2*
	Small subunit of ribosomal proteins (SSU)	rps2, rps3, rps4, rps7 *, rps8, rps11, rps12, rps14, rps15, rps16, rps18, rps19 *
	Large subunit of ribosomal proteins (LSU)	rpl2 *, rpl14, rpl16, rpl20, rpl22, rpl23 *, rpl32, rpl33, rpl36
Genes involved in photosynthesis	Subunits of NADH-dehydrogenase	ndhA, ndhB *, ndhC, ndhD, ndhE, ndhF, ndhG, ndhH, ndhI, ndhJ, ndhK
	Subunits in photosystem I	*psaA, psaB, psaC, psaI, psaJ*
	Subunits in photosystem II	*psbA, psbB, psbC, psbD, psbE, psbF, psbH, psbI, psbJ*, *psbK, psbL, psbM, psbN, psbT, psbZ*
	Subunits of cytochrome b/f complex	*petA, petB, petD, petG, petL, petN*
	Subunits of ATP synthase	*atpA, atpB, atpE, atpF, atpH, atpI*
	Large subunit of rubisco	*rbcL*
Other genes	Translational initiation factor	*infA*
	Protease	*clpP*
	Maturase	*matK*
	Subunit of Acetyl-CoA-carboxylase	*accD*
	Envelope membrane protein	*cemA*
	C-type cytochrome synthesis gene	*ccsA*
Conserved reading frames	Conserved open reading frames	ψrps19, ycf1 * (*ycf1, ψycf1*), ycf2 *, ycf3, ycf4, ψycf15

* Duplicated genes.

## Supplementary Materials

The following are available online at https://www.mdpi.com/2223-7747/9/11/1523/s1, Table S1: Voucher details and GenBank accession numbers of taxa used in this study, Table S2: The indexes of the codon usage bias in the *Hansenia*, *Haplosphaera* and *Sinodielsia* species.
