# Supplementary material for: Complete Plastid Genome Sequencing of Eight Species from Hansenia, Haplosphaera and Sinodielsia (Apiaceae): Comparative Analyses and Phylogenetic Implications
Source: Plants (Basel). 2020 Nov 9;9(11):1523. doi: 10.3390/plants9111523 (PMC7695273; doi:10.3390/plants9111523)
Supplement: Supplementary file 1 [file plants-09-01523-s001.pdf]

Table S1. Voucher details and GenBank accession numbers of taxa used in this study

Taxa	GenBank accession number	Voucher number	Locality
Angelica laxifoliata Diels	MH180258		
Bupleurum boissieuanum H.Wolff	MF663725		
Bupleurum chinense Franch.	MN893666		
Carum carvi L.	KR048286		
Chamaesium delavayi (Franch.) R.H.Shan & S.L.Liou	MN119367		
Chamaesium paradoxum H.Wolff	MK780227		
Chuanminshen violaceum M.L.Sheh & R.H.Shan	KU921430		
Cicuta virosa L.	KX352466		
Crithmum maritimum L.	HM596072		
Cryptotaenia japonica Hassk.	MK629764		
Hansenia forbesii (H.Boissieu) Pimenov & Kljuykov	MT834935*	J18081401(SZ)	Yuzhong, Gansu, China
Hansenia forrestii (H.Wolff) Pimenov & Kljuykov	MT843761*	J18102301(SZ)	Yajiang, Sichuan, China

Hansenia oviformis (R.H.Shan) Pimenov & Kljuykov	MT843762*	J19102201(SZ)	Shanyang, Shanxi, China
Hansenia weberbaueriana (Fedde ex H.Wolff) Pimenov & Kljuykov	MT843763*	J18081901(SZ)	Qilian, Qinghai, China
Haplosphaera himalayensis Ludlow	MK353674*	G18100802(SZ)	Nyingchi, Tibet, China
Haplosphaera phaea Hand.-Mazz.	MK801097	J18072109(SZ)	Deqin, Yunnan, China
Heracleum candicans Wall. ex DC.	MK522402		
Ligusticum chuanxiong S.H.Qiu, Y.Q.Zeng, K.Y.Pan, Y.C.Tang & J.M.Xu	KX594382		
Ligusticum delavayi Franch.	MT409613		
Pastinaca pimpinellifolia M.Bieb.	KM035850		
Peucedanum japonicum Thunb.	KU866530		
Pleurospermum camtschaticum Hoffm.	KU041142		
Prangos fedtschenkoi (Regel & Schmalh.) Korovin	KY652265		
Pterygopleurum neurophyllum (Maxim.) Kitag.	KT983257		
Seseli montanum L.	KM035851		
Sinodielsia microloba Kljuykov	MT843766*	F18 (SZ)	Nyingchi, Tibet, China

Sinodielsia yunnanensis H.Wolff (EY)	MT843765*	Y6 (SZ)	Eryuan, Yunnan, China
Sinodielsia yunnanensis H.Wolff (HB)	MK993275	K1(SZ)	Shangri-La, Yunnan, China
Sinodielsia yunnanensis H.Wolff (KM)	MT843764*	F10 (SZ)	Kunming,, Yunnan, China
Tongoloa silaifolia H.Wolff	NC_049062		
Torilis scabra DC.	MN105615		
Trachyspermum ammi Sprague	MN746303		

*: Newly generated sequences; otherwise, sequences were obtained from GenBank.

Table S2. The indexes of the codon usage bias in the *Hansenia*, *Haplosphaera* and *Sinodielsia* species.

Index	*Hansenia forbesii*	*Han. forrestii*	*Han. oviformis*	*Han. weberbaueriana*	*Haplosphaera himalayensis*	*Hap. phaea*	*~~Meeboldia~~Sinodielsia yunnanensis* (HB)	*~~M~~S. yunnanensis* (KM)	*S. yunnanensis* (EY)*~~Peucedanum delavayi~~*	*Sinodielsia microloba*
Codon No.	21,228	21,213	21,230	21,254	21,229	21,207	21,147	21,134	21,146	21,162
Amino acid No.	21,175	21,160	21,177	21,201	21,176	21,154	21,094	21,081	21,093	21,109
CAI	0.167	0.167	0.167	0.167	0.167	0.167	0.166	0.166	0.166	0.166
CBI	-0.098	-0.098	-0.099	-0.099	-0.1	-0.098	-0.101	-0.101	-0.101	-0.102
ENC	49.82	49.8	49.83	49.91	49.79	49.77	49.65	49.68	49.67	49.63
Fop	0.355	0.355	0.355	0.355	0.354	0.355	0.353	0.354	0.353	0.353
GC3s	0.271	0.27	0.271	0.271	0.269	0.27	0.266	0.267	0.267	0.266

CAI: Codon adaptation Index; CBI: Codon bias index; ENC: Effective number of codons; Fop: Frequency of optimal codons; GC3s: GC of silent 3rd codon position
